# Review of hydatid cyst cases and causes of recurrences complicating treatment, a 7-year cross-sectional study from Turkey: A single-center, retrospective observational study

**DOI:** 10.1097/MD.0000000000042861

**Published:** 2025-06-20

**Authors:** Nilgun Altin, Ali Acar, Onur Ergun, Semanur Kuzi, Tülay Ünver Ulusoy, Arif Doğan Habiloğlu, Ongay Kulirkin Kara

**Affiliations:** a Department of Infectious Diseases and Clinical Microbiology, Ankara Etlik City Hospital, Ankara, Turkey; b Department of Infectious Diseases and Clinical Microbiology, Bayindir Hospital Sogutozu, Ankara, Turkey; c Department of Radiology, Üsküdar University Faculty of Medicine, Istanbul, Turkey; d Department of Infectious Diseases and Clinical Microbiology, Batman Training and Research Hospital, Batman, Turkey.

**Keywords:** cystic echinococcosis, Gharbi classification, recurrence, treatment approach, zoonotic disease

## Abstract

Cystic echinococcosis (CE) is an endemic zoonotic disease in Turkey. Despite advances in treatment approaches, full eradication is not always possible. So, recurrence remains a major concern with a long-lasting silent-course necessitating close follow-up. Recurrence has higher morbidity and mortality requiring more complex treatment approaches. We aimed to evaluate the epidemiologic, laboratory data, treatment approaches, and their relationship with recurrence. This retrospective, cross-sectional study assessed patients with CE who were admitted to a training and research hospital between January 2015 and August 2021. Demographic, clinical characteristics, and laboratory findings of the patients were examined, and comparisons were made between those with and without recurrence. Multivariable logistic regression analysis was conducted to identify potential risk factors for CE recurrence. Of the total 214 patients with a mean age was 50.4 ± 16.5 years; 62.6% (n = 134) were female and 60.2 % (129) had a history of living in a rural area. The most common cyst localization was in the liver in 167 (78%). Although the diagnosis was confirmed by imaging methods in all patients, serological test positivity was found only in 72% (n = 140). Recurrence was seen in 17.8% (n = 38) of the patients, and recurrence most commonly occurred within the first 3 years in 25 patients (65.7%), 12 of whom (31.6%) experienced recurrence in the 1st year after treatment. The recurrence rate was higher in the patients with multiorgan involvement (odds ratios [OR] 2.5, 95% confidence interval [CI]: 1.1–5.6, *P* = .019), a hydatid cyst duration of more than 5 years (OR 5.5, 95% CI: 2.6–11.8, *P* < .001), and those who underwent invasive procedure (OR 4.6, 95% CI: 1.3–15.7, *P* = .014). Hydatid cyst duration > 5 years was an independent risk factor for the recurrence of CE (OR 3.5, 95% CI: 1.5–8.1, *P* = .003). CE is frequently seen in females, especially living in rural areas. It is difficult to diagnose with routine serological testing, so imaging techniques are needed for diagnosis. Although longer duration of the disease, multiorgan involvement, and invasive treatment were more in patients with recurrences, only the long duration of the disease was independently associated with recurrences. In addition, due to presence of very late recurrences, long-term follow-up is important.

## 1. Introduction

Human echinococcosis is a parasitic and zoonotic disease primarily caused by *Echinococcus granulosus.*^[1]^ Cystic echinococcosis (CE) is commonly observed in rural areas where contact with animals is frequent.^[1]^ Humans act as intermediate hosts and become infected by ingesting parasite eggs.^[2]^ CE can spread to multiple organs, progress through active and inactive phases, and is challenging to manage effectively.^[1]^ It is often diagnosed incidentally or after complications occur and is believed to have a much higher incidence than reported.^[3]^ The disease may remain asymptomatic for years until cysts grow large enough to cause symptoms. Up to 80% of patients have a single organ involved. In 70% to 80% of cases, cysts are found in the liver, 10% to 30% in the lungs, and 10% to 15% in the rest of the body. Multiorgan involvement is rare.^[4]^

The diagnosis of CE is based on epidemiological history, physical examination, imaging and serology. Imaging techniques such as ultrasound, and computed tomography are used to identify the structure of cysts and monitor patients during follow-up.^[2]^ Ultrasound surveys indicate that cysts may grow at a rate of 1 to 50 mm per year or remain unchanged for years. Cysts can also spontaneously rupture, collapse, or disappear. When diagnosis and effective treatment are delayed, the disease can lead to life-threatening complications and significant healthcare costs.^[4]^ Although mortality is rare (around 2–4%), it may increase significantly if treatment and care are inadequate. In cases of recurrence, treatment becomes more complex, with an elevated risk of morbidity and mortality.^[5]^

CE treatment is done according to the recommendations of the Informal Working Groups on Echinococcosis (WHO-IWGE). It considers factors such as cyst type, size, location the presence or absence of complications, and the availability of medical expertise and equipment.^[5,6]^ There are 4 therapeutic modalities for CE: chemotherapy, surgery, percutaneous drainage (puncture, aspiration, injection, reaspiration; PAIR), and “watch and wait” approach.^[2,6–8]^ Medical therapy is usually applied alongside surgery and percutaneous drainage. It may also be carried out as standalone treatment when patients are not candidates for other interventions.^[6]^ The primary goal of surgical treatment is to achieve complete elimination of the parasite and to prevent recurrence.^[9]^ Surgery remains the preferred treatment for patients with complicated cysts.^[2,3,10]^ Percutaneous drainage of hepatic hydatid cysts is now accepted as an alternative to surgery in the appropriate patients.^[2,3]^ However, recurrence can occur following any therapy.^[9]^ The intraoperative dissemination of protoscolex-rich hydatid fluid during surgery or the PAIR procedure is a major cause of recurrence.^[5]^ Surgeons frequently inject a protoscolecide into CE cysts as part of the invasive technique to reduce the risk of spillage and subsequent recurrence.^[5]^ Patients must be closely monitored after treatment, as recurrences remains a possibility.^[5,6]^

## 2. Aims and objectives

In this study, we aimed to evaluate the epidemiologic, laboratory data, and treatment approaches of patients admitted to our training and research hospital for 7 years with the diagnosis of cystic echinococcosis and to examine their relationship with recurrence.

## 3. Methods

### 3.1. Study population

This retrospective cross-sectional study assessed patients with a confirmed CE diagnosis who were admitted to our Training and Research Hospital from the city and surrounding provinces between January 2015 and August 2021. We included all consecutive patients without any restrictions except < 18 years old and incomplete diagnoses or treatment methods.

### 3.2. Data collection

The clinical, laboratory, and epidemiologic data of the patients in the given period were obtained retrospectively from electronic hospital records and by calling the patients by telephone. Data on age, gender, dog ownership, living in rural areas, disease duration, presence of chronic disease (including cancer, kidney disease, cardiovascular disease, stroke, diabetes mellitus) biochemical findings (aspartate aminotransferase [AST], alanine aminotransferase [ALT], alkaline phosphatase, gamma-glutamyl transpeptidase, bilirubin, albumin, leukocytes, lymphocytes, eosinophils, hemoglobin, hematocrit, mean corpuscular volume, red cells distribution width) were collected. Gharbi classification of CE, cyst localization, serological test results (indirect hemagglutination test [IHA]), treatment approaches, antihelminthic use and duration were also evaluated. AST/ALT, and new serological markers leukocyte/eosinophil and lymphocyte/eosinophil were calculated.

Gender, age, multiorgan involvements, chronic disease, invasive treatment processes, albendazole use, serologic positivity (IHA ≥ 1/160), hydatid cyst duration, AST, ALT, AST/ALT ratio, alkaline phosphatase, leukocytes, lymphocytes, eosinophils, lymphocyte/eosinophil ratio, leukocyte/eosinophil ratio, albumin, and Gharbi classification results were analyzed in relation to recurrence. Complete case analysis was conducted for analysis including Gharbi classification, as 44 patients had missing data.

### 3.3. Instruments

#### 3.3.1. CE diagnosis

Diagnosis were made according to the consensus report of the World Health Organization – Informal Working Group on Echinococcosis (WHO-IWGE), which is based on the detection of cysts by imaging methods and/or confirmation by serologic methods.^[6]^ Ultrasonography, computed tomography, and magnetic resonance imaging were used as imaging methods, and IHA was used as a serologic method. ELITechGroup (ELI.H.A Echinococcus-microbio-REF66604 lot9926) brand IHA kit was used to detect *E granulosus* specific antibodies.

#### 3.3.2. CE recurrence

After the procedure, ultrasonographic follow-up was performed every 3 months in the first year, every 6 months in the second year, and annually thereafter.^[11,12]^ Recurrent CE is defined as an increase in the diameter of the previously existing cyst(s) after treatment, radiological detection of viable cysts in the same or another localization, with or without an increase in titer levels in serological examinations.^[1]^

#### 3.3.3. Gharbi classification

According to the Gharbi classification based on ultrasound appearances, hepatic CE cysts were classified into 5 types.^[6,8,13]^

#### 3.3.4. CE treatment

Treatment decision is based on cyst type, size, location, and the presence or absence of complications.^[5,6]^ In our hospital, open surgery (packing) and/or percutaneous treatment (PAIR) is preferred as invasive procedure, depending on the patient’s medical condition and the localization of the cyst. Albendazole (10 mg/kg/day) was administered as an adjunct to invasive procedures, as well as to all patients in the medical treatment group. In the invasive treatment group, a hypertonic NaCl solution at a 20% to 30% concentration, was used in all cases.^[6,14]^

### 3.4. Ethical approval

This study was approved by the ethical review committee of our institution (September 20, 2021 – 120/09). The procedures followed in this study adhere to the tenets of the Declaration of Helsinki.

### 3.5. Data analysis

The data were evaluated using the SPSS 22.0 statistics package program. Continuous variables had normal distribution based on Kolmogorov–Smirnov test, therefore expressed as mean ± SD. Categorical variables were presented as frequency and percentages (%). The comparison of proportions of categorical variables was performed by chi-square or Fisher Exact test. Comparison of the continuous variables were performed using Student *t* test. Logistic regression analysis was conducted to identify potential risk factors for CE recurrence. Odds ratios (OR), 95% confidence intervals, and *P*-values were calculated for each potential predictor variable. In line with the conservative approach recommended by Hosmer, Lemeshow, and Sturdivant, variables with a *P*-value <.15 in univariate analysis were considered for the inclusion in the multivariate model to avoid prematurely excluding potentially important predictors.^[15]^ Multicolinearity was assessed by checking variance inflation factor, and no co-linearity was observed. All other *P*-values of <.05 were considered statistically significant.

## 4. Results

### 4.1. Demographic, epidemiologic, clinical and laboratory characteristics of the patients

Demographic, epidemiologic, clinical, and laboratory characteristics of the patients are presented in Table 1. Of the total 214 patients, the mean age was 50.4 ± 16.5 years, and 62.6% were female. The duration of the disease was 5.8 ± 5.5 (1–40) years. Thirty-five percent of cases have at least 1 chronic disease, with cardiovascular disease being the most common. Although the diagnosis was confirmed by imaging methods in all patients, serological tests were positive in only 140 (72 %) of the 194 patients who underwent testing. Sixty percent (n = 129) had a history of living in a rural area. The albendazole regimen varied according to the physicians’ discretion, but most commonly consisted of administration starting 1 week before and continuing for 3 months after the operation (n = 130, 60.8%). The most common cyst localization was in the liver (n = 167, 78%), followed by the lung (n = 33, 15%) and more than 1 location (n = 39, 18.2%). Patients were followed up for 4.8 ± 4.7 years (range; 0.5–34 years).

**Table 1:** Demographic, epidemiologic, clinical, and laboratory characteristics of the patients.

Age, years, mean ± SD (min–max)		50.4 ± 16.5 (18–89)
Female, n (%)		134 (62.6)
Underlying chronic disease, n (%)		75 (35.0)
Hydatid cyst duration, years, mean ± SD (min–max)		5.8 ± 5.5 (1–40)
IHA positivity (≥1/160), n (%)		140 (72)
AST, U/L, mean ± SD (min–max)		24.7 ± 17.5 (8.0–130.0)
ALT, U/L, mean ± SD (min–max)		28.0 ± 45.3 (5.0–447.0)
AST/ALT, mean ± SD (min–max)		1.2 ± 0.7 (0.3–8.1)
ALP, IU/l, mean ± SD (min–max)		103.1 ± 93.2 (30–944)
GGT, U/L, mean ± SD (min–max)		53.3 ± 88.2 (9–690)
Leukocyte, µL, mean ± SD (min–max)		8376.8 ± 3533.5 (2690–29,000)
Eosinophil, µL, mean ± SD (min–max)		353.5 ± 649.3 (0–5200)
Lymphocyte, µL, mean ± SD (min–max)		2135.4 ± 697.7 (700–6200)
Leukocyte/eosinophil, mean ± SD (min–max)		61.5 ± 87.8 (2.4–823)
Lymphocyte/eosinophil, mean ± SD (min–max)		16.2 ± 18.4 (0.4–199.0)
RDW, mean ± SD (min–max)		14.6 ± 2.8 (11.4–35.3)
MCV, mean ± SD (min–max)		83.5 ± 8.2 (29.0–97.1)
Hb, g/dl, mean ± SD (min–max)		13.4 ± 2.0 (7.6–18.1)
Hct, mean ± SD (min–max)		40.5 ± 5.5 (20.5–53.4)
Albumin, g/dL, mean ± SD (min–max)		4.1 ± 0.6 (1.0–5.9)
Treatment type, n (%)	Surgery	89 (41.6)
	PAIR	99 (46.3)
	Albendazole	214 (100)
Duration of albendazole treatment, n (%)	0–3 months	130 (60.8)
	4–6 months	50 (23.4)
	7–12 months	9 (4.2)
	>12 months	25 (11.7)
Owning a dog, n (%)		22 (10.3)
History of living in a rural area, n (%)		129 (60.2)
Currently, n (%)		58 (27.1)
In the past, n (%)		71 (33.2)

ALP = alkaline phosphatase, ALT = alanine aminotransferase, AST = aspartate aminotransferase, GGT = gamma-glutamyl transpeptidase, Hb = hemoglobin, Hct = hematocrit, IHA = indirect hemagglutination test, MCV = mean corpuscular volume, PAIR = puncture aspiration injection reaspiration, RDW = red cell distribution width, SD = standard deviation.

### 4.2. Recurrences

**Figure 1.**
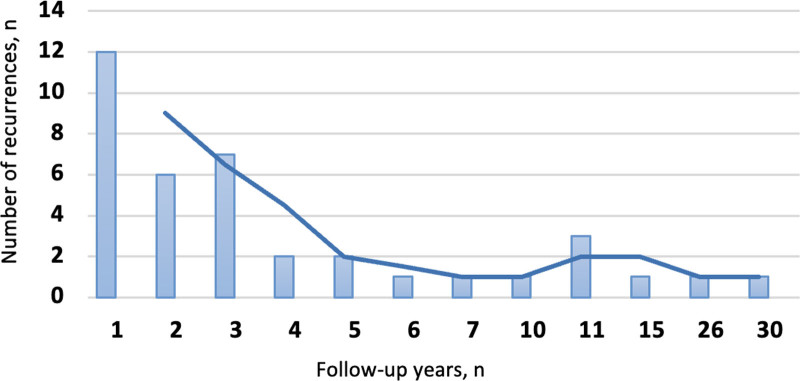
Distribution of recurrences according to years.

Recurrences were seen in 38 (17.8%) of the entire population after a mean duration of 5 ± 1.06 years (range: 1–30 years), with most occurring within the first 3 years. Of these, 25 patients (65.7%) experienced recurrence in the first 3 years, 12 of whom (31.6%) had recurrence within the first year after treatment (Fig. 1).

Surgery was performed in 89 patients (41.6%), among whom 27 (12.6%) experienced recurrence. PAIR was performed in 99 patients (46.3%), among whom 25 (11.7%) experienced recurrence. Of the 72 patients (33.6%) who underwent only PAIR, 8 (3.7%) had recurrence, while 62 patients (29%) who underwent only surgery had 10 recurrences (4.7%).

Gharbi classification was missing in 44 of our cases, and the typing of 170 classified cases and recurrence rates with cyst types and treatments are given in Table 2. Type 5 was the most common form (n = 45, 23.6%), while recurrence was most common in type 4 (Table 2).

**Table 2:** Distribution of recurrences according to Gharbi classification subtypes.

Gharbi classification	Type 1	Type 2	Type 3	Type 4	Type 5
n (%)	20 (10.5)	31 (16.2)	35 (18.3)	39 (20.4)	45 (23.6)
Total recurrence, n (%)	3 (1.6)	6 (3.1)	6 (3.1)	8 (4.2)	5 (2.6)
Recurrence, surgery, n (%)	3 (1.6)	4 (2.1)	3 (1.6)	4 (2.1)	4 (2.1)
Recurrence, PAIR, n (%)	3 (1.6)	6 (3.1)	2 (1.0)	6 (3.1)	2 (1.0)
Recurrence, only albendazole treatment, n (%)	0 (0.0)	0 (0.0)	1 (2.8)	1 (2.8)	1 (2.8)

PAIR = puncture aspiration injection reaspiration.

### 4.3. Risk factors for recurrences

Patients were grouped according to the presence of recurrences, and compared based on their demographic, clinical characteristics, and laboratory findings.

#### 4.3.1. Univariable analysis

On univariable analysis, recurrence was statistically higher in patients with multiorgan involvement (*P* = .019), those who underwent invasive procedure (*P* = .014), and those with hydatid cyst duration of more than 5 years (*P* < .001). However, no statistical difference was detected between patients with and without recurrence in terms of age, gender, presence of chronic disease, serological test positivity (IHA ≥ 1/160), lymphocyte/eosinophil and leukocyte/eosinophil ratios, AST/ALT ratios, other laboratory findings, cyst localization (hepatic or extrahepatic), or cyst type according to the Gharbi classification (Table 3).

**Table 3:** Univariable logistic regression analyses for the recurrence of cystic echinococcosis (CE).

	Recurrence n = 38	Non-recurrence n = 176	Univariable analysis	
			OR (95% CI)	*P*
Age, mean ± SD	51.7 ± 17.0	50.1 ± 16.5	1.0 (0.9–1.0)	.60
Female, n (%) Male, n (%)	27 (20.2) 11 (13.8)	107 (79.9) 69 (86.3)	1.0 (0.5–1.8)	.236
Multiorgan involvement, n (%)	12 (30.8)	27 (69.2)	**2.5 (1.1–5.6**)	**.019**
Extrahepatic location, n (%)	9 (19.2)	38 (80.9)	1.0 (0.4–2.4)	.658
Presence of chronic disease, n (%)	15 (20.0)	60 (80.0)	1.2 (0.6–2.5)	.154
Invasive treatment vs albendazole, n (%)	35 (21.7)	126 (78.3)	**4.6 (1.3–15.7**)	**.014**
IHA ≥ 1/160 n (%)	31 (22.1)	109 (77.9)	2.1 (0.8–5.5)	.106
Hydatid cyst duration/year (mean ± SD)	10.84 (±9.23)	4.69 (±3.37)	**5.5 (2.6–11.8**)	**<.001**
Gharbi classification (n, %)	28 (17.5)	142 (82.5)	1.3 (0.2–6.1)	.765
Laboratory findings (mean ± SD)				
AST, U/L	21.67 (±7.43)	35.28 (±18.92)	0.9 (0.9–1.0)	.257
ALT, U/L	23.11 (±17.72)	29.04 (±49.11)	0.9 (0.9–1.0)	.470
AST/ALT	1.14 (±0.50)	1.18 (±0.69)	0.9 (0.5–1.6)	.786
ALP, IU/L	84.92 (±30.75)	107.15 (±101.61)	0.9 (0.9–1.0)	.190
GGT, U/L	42.18 (±47.70)	55.72 (±94.84)	0.9 (0.9–1.0)	.400
Leukocyte/µL	7698.95 (±2010.64)	8523.18 (±3770.98)	0.9 (0.9–1.0)	.193
Lymphocyte/µL	2180 (±634.30)	2125 (±711.99)	1.0 (0.9–1.0)	.665
Eosinophil/µL	288.86 (±302.71)	367.47 (±702.17)	0.9 (0.9–1.0)	.517
Lymphocyte/eosinophil	13.52 (±10.41)	16.72 (±19.64)	0.9 (0.9–1.0)	.356
Leukocyte/eosinophil	44.38 (±31.35)	65.19 (±95.27)	0.9 (0.9–1.0)	.210
Albumin, g/dL	4.20 (±0.57)	4.05 (±0.65)	1.5 (0.8–2.9)	.194

Bold values indicate statistically significant results.

ALP = alkaline phosphatase, ALT = alanine aminotransferase, AST = aspartate aminotransferase, CI = confidence interval, GGT = gamma-glutamyl transpeptidase, IHA = indirect hemagglutination test, OR = odds ratio, SD = standard deviation.

#### 4.3.2. Multivariable analysis

Variables with a *P*-value < .15 in univariable analysis were included in multivariable logistic regression analysis and independent risk factors for recurrence were evaluated (Table 4). Only hydatid cyst duration of longer than 5 years was independently associated with recurrence (OR3.5, CI 1.5–8.1, *P* = .003).

**Table 4:** Multivariable logistic regression model for the occurrence of recurrence in cystic echinococcosis (CE).

Variables	Multivariable analysis results OR (95% CI), *P*	
Invasive procedure vs only albendazole	2.7 (0.7–10.2)	.131
IHA ≥ 1/160	2.8 (0.9–8.4)	.058
Hydatid cyst duration > 5 years	3.5 (1.5–8.1)	**.003**
Multiple organ involvement	2.0 (0.8–5.0)	.136

Bold value indicates statistically significant results.

IHA = indirect hemagglutination test.

## 5. Discussion

In this study, the demographic, clinical characteristics, and laboratory findings of patients diagnosed with CE were evaluated, risk factors for recurrences were analyzed. In short, the majority of patients were female, lived in rural areas, most common localization of CE was in the liver, and recurrence rate was 17.8% occuring mostly in the first year after treatment. Although recurrence of CE was statistically higher in patients with multiorgan involvement, those who underwent invasive procedures, and those with hydatid cyst duration of more than 5 years, only hydatid cyst duration > 5 years was an independent risk factor.

Most patients with CE were women. It is widely accepted that sex hormones, sex chromosomes, genomic, and epigenetic differences can contribute to variations in the immune responses between women and men.^[16]^

Socioeconomic and cultural characteristics are among the best defined risk factors for human infection: dogs living closely with people, uncontrolled slaughter of livestock, and unsanitary living conditions.^[2]^ These may be responsible for the higher prevalence of the disease in rural areas.

Although theoretically, echinococcosis can affect any organ, liver is the most frequently affected organ, followed by the lungs.^[17,18]^ Similarly, liver involvement was found in 78% (n = 167), lung involvement in 15% (n = 33), and multiorgan involvement in 18% (n = 39) of the cases in our study.

In our study, recurrence rate was found to be 17.8%, that is within the reported range of previous studies’ recurrence rates of 3.4% to 25%.^[19–26]^ This wide range was explained by the heterogenities in the pre- and postoperative medical treatment, number and size of cysts, localization, Gharbi classification, and surgical or percutaneous procedures performed.^[20–26]^ Aydin et al, conducted a study in which they performed radical surgery in 92 patients and conservative surgery in 129 patients and reported the recurrence rates as 3.2 % and 24 %, respectively.^[20]^ Yagci et al, reported recurrence rates as 16.2%, 3.3%, and 3.5% after open surgery, laparoscopic surgery, and percutaneous treatment, respectively.^[25]^ Çetinçakmak et al, reported recurrence rates of 6.7% after percutaneous procedures.^[26]^ In our study, invasive treatment and multiorgan involvement were associated with higher recurrence rates, although neither of them were independent risk factors. Microscopic spillage of live parasites during surgery, failure to remove all viable cysts in inaccessible or difficult areas, or leaving a cyst wall residue in the first operation are considered the most common causes of recurrences.^[9]^ Also, the need for invasive procedures is generally higher in cases with multiple localizations.^[9]^ This may explain why the need for multiple and challenging invasive procedures in multiorgan involvement may be an important risk factor for the development of recurrences, as in our cases.

Another major finding of the present study is the longer the disease, the higher the incidence of recurrences. Up to our knowledge, the relationship of the disease duration and recurrences were not reported. Especially, hydatid cyst disease of the liver and lung, in which it is most frequently located, may grow asymptomatically to large sizes before clinical signs and symptoms develop, in contrast to the cysts of other organs such as brain, heart, bone, and eye.^[2]^ In addition to this preclinical disease duration, the summation of the duration after clinical signs and symptoms develop, the total disease duration may be higher than predicted. As a logical explanation, longer duration, which makes the hematological dissemination easier, leads to multiorgan involvement more possible, which in turn makes the invasive procedures to completely eliminate the disease more difficult, and hence increases post-procedure recurrences.

Recurrences can be detected 3 months to 20 years after surgery, although mostly accumulated to the first 3 years after treatment, in concordance with our results.^[9,24]^ Confirmation of therapeutic efficacy is difficult, as relapses can occur years later.^[9]^ So it is recommended that the follow-up period should not be shorter than 5 years after surgical procedures.^[5]^ The onset of CE is often asymptomatic, and even clinical evaluation, supported by serologic testing, and serologic tests can be non-diagnostic, as in our study in which the diagnostic yield of serology was only 72%. Therefore, follow-up with ultrasonography should be performed every 3 months in the first year, every 6 months in the second year, and annually in the following years for at least 3 years.^[9,21,27]^ Considering that recurrences in our cases were usually seen within the first 3 years and the mean duration was 5 years.

Preoperative medical treatment with albendazole is also recommended by the World Health Organization (WHO) because it reduces recurrences and facilitates surgery by reducing intracyst pressure.^[12,28]^ In a study of 63 patients by Bülbüller et al, albendazole was used in 51% of preoperative patients and its effect on recurrences was not found to be significant.^[29]^ In our study, all the patients received albendazole.

Many studies in the literature indicate that there is no relationship between Gharbi classification and cyst type and recurrence.^[9,21,29,30]^ In a recent study by Temiz et al, the majority of cysts were type 3 according to the Gharbi classification and were not associated with recurrence.^[21]^ In the study by Tirado et al, recurrence was more common in type 2 to 3 than in type 4 to 5, and recurrence was higher in those with 2 or more cysts, but it was not statistically significant.^[30]^ In our study, although the majority of recurrent cysts (72%) were Gharbi type 2, 3, and 4; it was not statistically significant.

## 6. Limitations of study

The study has certain limitations: first, as this study has a retrospective design and follow-up duration is very long for some patients; we cannot exclude selection bias and recall bias. Secondly, this was a single-center study, which may not accurately reflect the results of other centers across the country. Third, among the epidemiologic data of the patients, information on occupation, educational status, social environment, and economic level were missing. Fourth, the size of the cysts was not available in our study. Finally, 44 patients had missing data for Gharbi classification, however, we conducted complete case analysis for the analysis including Gharbi classification. Prospective multicenter studies are needed to provide more robust outcome evidence. On the other hand, our study has some strengths such as this study includes one of the largest patient populations, and also we recruited all the patients in a given period.

## 7. Conclusion

CE is frequently seen in females, especially living in rural areas. It is difficult to diagnose with routine serological testing and imaging techniques are needed for confirmation of diagnosis. Although longer duration of the disease, multiorgan involvement, and invasive treatment were more in patients with recurrences, only the long duration of the disease was independently associated with recurrences after treatment. In addition, due to presence of very late recurrences, long-term follow-up of is important.

## Author contributions

**Conceptualization:** Nilgun Altin, Tülay Ünver Ulusoy.

**Data curation:** Nilgun Altin, Onur Ergun, Semanur Kuzi, Ongay Kulirkin Kara.

**Formal analysis:** Nilgun Altin, Ali Acar.

**Investigation:** Nilgun Altin, Arif Doğan Habiloğlu.

**Writing – original draft:** Nilgun Altin.

**Writing – review & editing:** Ali Acar, Onur Ergun, Arif Doğan Habiloğlu.

## References

[1] CDC. DPDx – Echinococcosis laboratory identification of parasites of public health concern. 2019. https://www.cdc.gov/dpdx/echinococcosis/index.html.

[2] Coyle CM. Echinococcosis: cystic and alveolar disease. Netter’s Infect Dis. 2012:491–501. https://einstein.elsevierpure.com/en/publications/echinococcosis-cystic-and-alveolar-disease.

[3] Borhani M Fathi S Darabi E. Echinococcoses in Iran, Turkey, and Pakistan: old diseases in the new millennium. Clin Microbiol Rev. 2021;34:e0029020. 34076492 10.1128/CMR.00290-20 PMC8262809

[4] Duran E Pehlivan B. Factors increasing mortality in Echinococcosis patients treated percutaneously or surgically. A review of 1,143 patients: a retrospective single center study. Eur Rev Med Pharmacol Sci. 2023;27:493–500. 36734716 10.26355/eurrev_202301_31049

[5] Wen H Vuitton L Tuxun T. Echinococcosis: advances in the 21st century. Clin Microbiol Rev. 2019;32:e00075–18. 30760475 10.1128/CMR.00075-18 PMC6431127

[6] Brunetti E Kern P Vuitton DA. Expert consensus for the diagnosis and treatment of cystic and alveolar echinococcosis in humans. Acta Trop. 2010;114:1–16. 19931502 10.1016/j.actatropica.2009.11.001

[7] Agudelo Higuita NI Brunetti E McCloskey C. Cystic echinococcosis. J Clin Microbiol. 2016;54:518–23. 26677245 10.1128/JCM.02420-15 PMC4767951

[8] WHO Informal Working Group. International classification of ultrasound images in cystic echinococcosis for application in clinical and field epidemiological settings. Acta Trop. 2003;85:253–61. 12606104 10.1016/s0001-706x(02)00223-1

[9] El Malki HO El Mejdoubi Y Souadka A. Does primary surgical management of liver hydatid cyst influence recurrence? J Gastrointest Surg. 2010;14:1121. 20464525 10.1007/s11605-010-1220-0

[10] Dogru MV Sezen CB Aker C. Evaluating giant hydatid cysts: factors affecting mortality and morbidity. Ann Thorac Cardiovasc Surg. 2020;27:164–8. 33162437 10.5761/atcs.oa.20-00178 PMC8343032

[11] Akinci D. Drenaj ve Skleroterapi. Turkiye Klinikleri J Radiol-Special Topics. 2012;5:19–25.

[12] Guidelines for treatment of cystic and alveolar echinococcosis in humans. WHO Informal Working Group on Echinococcosis. Bull World Health Organ. 1996;74:231–42. 8789923 PMC2486920

[13] Gharbi HA Hassine W Brauner MW Dupuch K. Ultrasound examination of the hydatic liver. Radiology. 1981;139:459–63. 7220891 10.1148/radiology.139.2.7220891

[14] Sharafi SM Sefiddashti RR Sanei B Yousefi M Darani HY. Scolicidal agents for protoscolices of *Echinococcus granulosus* hydatid cyst: review of literature. J Res Med Sci. 2017;22:92. 28900448 10.4103/jrms.JRMS_1030_16 PMC5583616

[15] Hosmer DW Jr Lemeshow S Rodney X. Sturdivant. 3rd Ed. Applied Logistic Regression; 2013.

[16] Piasecka B Duffy D Urrutia A. Distinctive roles of age, sex, and genetics in shaping transcriptional variation of human immune responses to microbial challenges. Proc Natl Acad Sci U S A. 2018;115:E488–97. 29282317 10.1073/pnas.1714765115 PMC5776984

[17] Baruah A Sarma K Barman B. Clinical and laboratory presentation of hydatid disease: a study from Northeast India. Cureus. 2020;12:e10260. 33042698 10.7759/cureus.10260 PMC7537570

[18] Conchedda M Antonelli A Caddori A Gabriele F. A retrospective analysis of human cystic echinococcosis in Sardinia (Italy), an endemic Mediterranean region, from 2001 to 2005. Parasitol Int. 2010;59:454–9. 20601105 10.1016/j.parint.2010.06.008

[19] Aydin U Yazici P Onen Z. The optimal treatment of hydatid cyst of the liver: radical surgery with a significant reduced risk of recurrence. Turk J Gastroenterol. 2008;19:33–9. 18386238

[20] Aydin U Çoker A. The optimal treatment of hydatid cyst of the liver: radical surgery with a significant reduced risk of recurrence. Turk J Gastroenterol. 2008;19:33–9. 18386238

[21] Temiz A Öztürk G Kisaoğlu A. Factors related to recurrence in surgical treatment of hydatid cyst. Arch Clin Exp Med. 2018;3:1–2.

[22] Şahin DA Kuşaslan R Türel KS. Surgical treatment in our hydatid cyst patients and efficiency of sphincterotomy with ERCP (Karaciğer Kist Hidatik Olgularimizda Cerrahi Tedavi ve ERCP ile Sfinkterotominin Etkinliği). Med J Kocatepe. 2006;7:11–6.

[23] Yorganci K Sayek I. Surgical treatment of hydatid cysts of the liver in the era of percutaneous treatment. Am J Surg. 2002;184:63–9. 12135724 10.1016/s0002-9610(02)00877-2

[24] Kapan M Kapan S Goksoy E Perek S Kol E. Postoperative recurrence in hepatic hydatid disease. J Gastrointest Surg. 2006;10:734–9. 16713547 10.1016/j.gassur.2005.10.013

[25] Yagci G Ustunsoz B Kaymakcioglu N. Results of surgical, laparoscopic, and percutaneous treatment for hydatid disease of the liver: 10 years experience with 355 patients. World J Surg. 2005;29:1670–9. 16311852 10.1007/s00268-005-0058-1

[26] Deniz MA Taş Deniz Z Hattapoğlu S Çetinçakmak MG. Retrospective evaluation of percutaneous treated liver cyst hydatic cases (Perkütan Tedavi Edilmiş Karaciğer Kist Hidatik Olgularinin Retrospektif Değerlendirilmesi). Van Med J. 2020;27:267–73.

[27] Prousalidis J Kosmidis C Anthimidis G Kapoutzis K Karamanlis E Fachantidis E. Postoperative recurrence of cystic hydatidosis. Can J Surg. 2012;55:15–20. 21939605 10.1503/cjs.013010 PMC3270079

[28] Wani RA Malik AA Chowdri NA Wani KA Naqash SH. Primary extrahepatic abdominal hydatidosis. Int J Surg. 2005;3:125–7. 17462273 10.1016/j.ijsu.2005.06.004

[29] Bülbüller N Ilhan YS Kirkil C Yeniçerioğlu A Ayten R Cetinkaya Z. The results of surgical treatment for hepatic hydatid cysts in an endemic area. Turk J Gastroenterol. 2006;17:273–8. 17205405

[30] Velasco-Tirado V Romero-Alegría A Belhassen-García M. Recurrence of cystic echinococcosis in an endemic area: a retrospective study. BMC Infect Dis. 2017;17:455. 28655301 10.1186/s12879-017-2556-9 PMC5488421

